# Computational Evidence for Digenic Contribution of *AIPL1* and *BBS2* Rare Variants in Inherited Retinal Dystrophy

**DOI:** 10.3390/ijms26199430

**Published:** 2025-09-26

**Authors:** Simona Alibrandi, Concetta Scimone, Giorgia Abate, Sergio Zaccaria Scalinci, Antonina Sidoti, Luigi Donato

**Affiliations:** 1 Department of Biomedical and Dental Sciences and Morphofunctional Imaging, University of Messina, Via Consolare Valeria 1, 98125 Messina, Italy; simona.alibrandi@unime.it (S.A.); giorgia.abate@studenti.unime.it (G.A.); asidoti@unime.it (A.S.); ldonato@unime.it (L.D.); 2 Department of Biomolecular Strategies, Genetics, Cutting-Edge Therapies, I.E.ME.S.T., Via Michele Miraglia 20, 90139 Palermo, Italy; 3 Department of Medical and Surgical Sciences, University of Bologna, 40121 Bologna, Italy; sergio.scalinci@unibo.it

**Keywords:** inherited retinal dystrophy, oligogenic mechanisms, *AIPL1*, *BBS2*, photoreceptor proteostasis, ciliary trafficking

## Abstract

Inherited retinal dystrophies (IRDs) are clinically and genetically heterogeneous disorders. Most IRDs follow a monogenic inheritance pattern. However, an increasing number of unresolved cases suggest the possible contribution of oligogenic or digenic mechanisms. Here, we report two ultra-rare missense variants—AIPL1 R302L and BBS2 P134R—that co-segregate with early-onset nonsyndromic retinal degeneration in affected individuals from a non-consanguineous family. We performed a multi-level computational investigation to assess whether these variants may act through a convergent pathogenic mechanism. Using AlphaFold2-predicted structures, we modeled both wild-type and mutant proteins, introduced point mutations, and performed energy minimization and validation. FoldX, DynaMut2, and DUET all predicted destabilizing effects at the variant sites, corroborated by local disruption of secondary structure and altered surface electrostatics. Comparative docking (via HDOCK and ClusPro) identified a putative interaction interface between the TPR domain of AIPL1 and the β-sheet face of BBS2. This interface was destabilized in the double-mutant model. At the systems level, transcriptomic profiling confirmed co-expression of AIPL1 and BBS2 in human retina and fetal eye, while functional enrichment analysis highlighted overlapping involvement in ciliary and proteostasis pathways. Network propagation suggested that the two proteins may converge on shared interactors relevant to photoreceptor maintenance. Collectively, these in silico results provide structural and systems-level support for a candidate digenic mechanism involving *AIPL1* and *BBS2*. While experimental validation remains necessary, our study proposes a testable mechanistic hypothesis and underscores the value of computational approaches in uncovering complex genetic contributions to IRDs.

## 1. Introduction

Inherited retinal dystrophies (IRDs) comprise a group of clinically and genetically diverse disorders that primarily affect the structure and function of photoreceptors. These conditions are among the leading causes of early-onset vision loss and blindness in children and young adults. IRDs can follow autosomal recessive, autosomal dominant, X-linked, or non-Mendelian forms such as digenic or oligogenic inheritance. Understanding the molecular basis of IRDs is crucial for the development of accurate diagnostic strategies and targeted therapies [1].

Two genes of particular interest in the context of early-onset IRDs are *AIPL1* and *BBS2*. While functionally distinct, both are critical for photoreceptor homeostasis and are strongly implicated in cilia-related biology within the retina [2].

The *AIPL1* gene encodes aryl hydrocarbon receptor-interacting protein-like 1, a retina-specific molecular chaperone essential for the correct folding and stability of phosphodiesterase 6 (PDE6), the effector enzyme of the phototransduction cascade [3]. PDE6 is localized specifically in the photoreceptor outer segment, where it is transported via intraflagellar transport (IFT) mechanisms and the BBSome complex [4]. AIPL1 ensures optimal conformation and enzymatic orientation of PDE6 within this specialized ciliary structure. AIPL1 contains an N-terminal FK506-binding protein (FKBP)-like domain and a C-terminal tetratricopeptide repeat (TPR) domain, both of which are required for interaction with PDE6 subunits and regulatory elements such as PDE6γ [5,6]. In photoreceptors, AIPL1 ensures the production of catalytically active holoenzyme complexes and prevents the accumulation of misfolded intermediates. Loss-of-function mutations in *AIPL1* result in rapid photoreceptor degeneration due to unregulated cGMP levels, ultimately leading to Leber congenital amaurosis type 4 (LCA4), one of the most severe IRD subtypes with onset at birth or early infancy [7].

*BBS2*, on the other hand, encodes a structural component of the BBSome, a multi-protein complex responsible for cargo trafficking within primary cilia, including the photoreceptor connecting cilium. The BBSome acts as an adaptor for intraflagellar transport (IFT) complexes and is essential for the compartmentalization and dynamic turnover of outer segment disks [8]. Mutations in *BBS2* are classically associated with Bardet–Biedl syndrome (BBS), a multisystem ciliopathy characterized by retinitis pigmentosa, obesity, polydactyly, renal anomalies, and cognitive impairment. However, recent reports have identified *BBS2* missense variants—including P134R—in patients with non-syndromic retinitis pigmentosa, suggesting a context-dependent or hypomorphic contribution of *BBS2* to retinal disease phenotypes [9].

The possibility of functional convergence between AIPL1 and BBS2 arises from their mutual involvement in cilia-dependent photoreceptor functions. AIPL1 activity ensures the structural integrity and enzymatic competence of PDE6, which is localized in the outer segment—a highly specialized ciliary compartment. Simultaneously, the BBSome, with BBS2 as a core subunit, is required for the biogenesis and continual renewal of this compartment [8]. Thus, pathogenic variants in both genes may contribute synergistically to retinal degeneration by disrupting distinct but converging cellular processes: protein folding and ciliary trafficking [10].

From a clinical perspective, recognizing digenic inheritance mechanisms in IRDs is essential for accurate molecular diagnosis and counseling. Digenic interactions may underlie cases that remain unsolved under a monogenic framework, especially in families with atypical segregation or variable expressivity. Incorporating such models into diagnostic pipelines can improve the interpretation of rare variants and support personalized management strategies in inherited retinal disorders.

This study was prompted by the identification of two rare missense variants, AIPL1 R302L and BBS2 P134R, in a family with inherited retinal dystrophy showing phenotypic variability among affected individuals. AIPL1 R302L is likely the primary disease-causing variant, while BBS2 P134R may act as a modifier based on conservation, structural effects, and prior reports. Given the emerging evidence for digenic or oligogenic mechanisms in IRDs, we aimed to evaluate whether these two variants act in concert to explain the observed non-Mendelian inheritance and phenotypic diversity.

Prompted by the identification of two rare missense variants in *AIPL1* and *BBS2* in individuals with early-onset nonsyndromic retinal degeneration, this study aimed to evaluate the potential for functional convergence between these two genes in the context of IRDs. We employed a multi-step in silico approach to model the structural and bioenergetic impact of each variant, assess their potential for direct or indirect physical interaction, and examine their involvement in shared retinal pathways. By integrating molecular modeling, docking simulations, and network-based functional analyses, we sought to determine whether the co-occurrence of *AIPL1* R302L and *BBS2* P134R could plausibly support a digenic inheritance model underlying the observed phenotype. Rather than establish definitive causality, our goal was to generate a mechanistic hypothesis consistent with the clinical and genetic data, and to provide a rationale for further experimental validation.

## 2. Results

### 2.1. Genetic Findings and Variant Prioritization

WES identified two rare heterozygous missense variants across selected patients: one located in the *AIPL1* gene (NM_014336.4:c.905G>T), causing an arginine-to-leucine substitution at residue 302 (p.Arg302Leu), and the other in the *BBS2* gene (NM_031885.3:c.401C>G), resulting in a proline-to-arginine substitution at residue 134 (p.Pro134Arg). Both variants affect highly conserved amino acid residues critical for protein structure and function and were validated by targeted Sanger sequencing.

In each family where segregation analysis was feasible, co-segregation of the two variants with the retinal dystrophy phenotype was confirmed. Specifically, all affected individuals were double heterozygotes for the identified variants (*AIPL1*^R302L/+^; *BBS2*^P134R/+^), whereas unaffected relatives carried either a single heterozygous variant or none. These findings support a model of potential digenic inheritance with variable penetrance.

Population frequency data retrieved from gnomAD v3.1.2 and ExAC databases indicated the extreme rarity of both variants (MAF < 1.0 × 10^−5^) and absence of homozygotes. Neither variant is classified as definitively pathogenic in ClinVar or HGMD Professional (Table 1), although BBS2 p.Pro134Arg has been previously reported in non-syndromic retinitis pigmentosa as a potential hypomorphic allele in double heterozygosity [9].

Other variants identified by WES were classified as benign or VUS and excluded based on lack of functional or clinical evidence. The evolutionary origin of *AIPL1* and *BBS2* variants remains undetermined due to insufficient haplotype data.

Taken together, these findings suggest a genetically heterogeneous but convergent scenario, where the co-occurrence of *AIPL1* and *BBS2* missense variants across distinct familial backgrounds contributes to a consistent retinal phenotype, supporting a potential digenic mechanism of disease.

### 2.2. Structural Modeling and Bioenergetic Impact

#### 2.2.1. Structural Consequences of AIPL1 R302L

The AIPL1 protein is composed of a 384-amino acid polypeptide featuring an N-terminal FKBP-like peptidyl-prolyl isomerase domain and a C-terminal TPR domain comprising three canonical motifs (TPR1–3) [11]. Structural modeling using the AlphaFold2-predicted structure (AF-Q9NZN9-F1) with a pLDDT of 91 for the TPR region revealed that Arg302 is located at the base of the third TPR motif (TPR3), positioned within a helix-turn-helix interface critical for PDE6γ subunit recognition [12].

**Figure 1:**
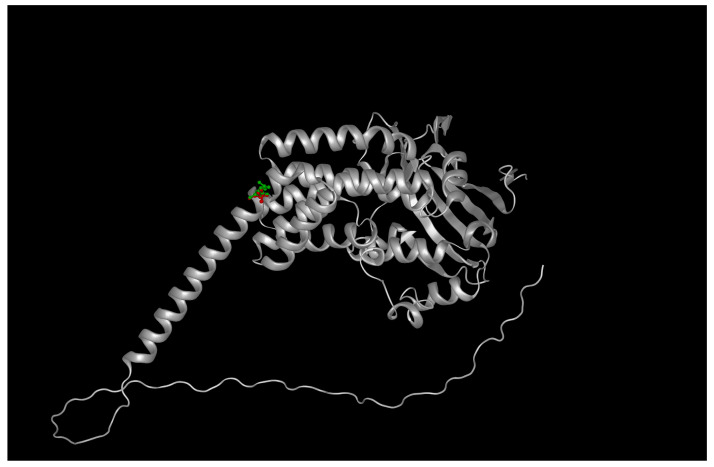
Structural localization and evolutionary conservation of the AIPL1 R302L variant. The AlphaFold2-predicted model shows Arg302 positioned at the apex of the TPR3 domain within a high-confidence region (pLDDT > 90). The visible C-terminal tail corresponds to a low-confidence, intrinsically disordered region (pLDDT < 50), included here for completeness but excluded from all stability, docking, and electrostatic analyses.

Substitution of the charged, flexible arginine with a hydrophobic leucine introduces both steric and electrostatic perturbations. PyMOL-based mutagenesis and ChimeraX side-chain optimization showed a loss of three hydrogen bonds formerly formed by the guanidinium group of R302 with backbone carbonyls of adjacent helices (residues Glu299 and Asp305). Electrostatic surface potential mapping using APBS confirmed a localized loss of positive charge near the protein surface, which may impair electrostatic complementarity with PDE6 subunits [13] (Figure 1, see Supplementary Tables S2 and S3).

Energetic modeling using FoldX5 predicted a destabilization of +2.14 kcal/mol (ΔΔG) [14], while DynaMut2 simulation incorporating ENCoM-derived flexibility changes revealed a local gain in atomic fluctuations centered at residues 298–308 as showed in Table 2.

This increase in local entropy is consistent with impaired structural rigidity of the TPR groove, a known determinant of AIPL1’s co-chaperone specificity [15].

**Figure 2:**
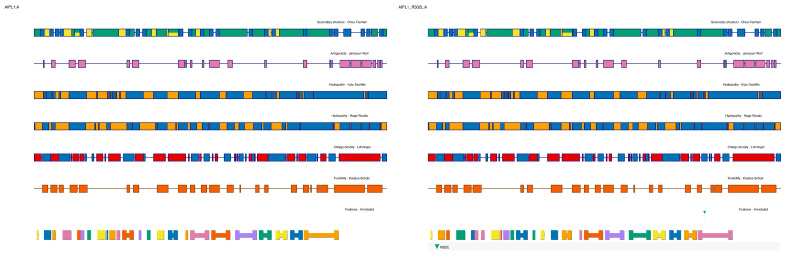
Structural and energetic effects of the AIPL1 R302L substitution. The R302L mutation disrupts polar contacts and increases local flexibility within the TPR3 domain. FoldX modeling predicts thermodynamic destabilization (ΔΔG = +2.14 kcal/mol), indicating impaired structural integrity.

Hydropathy plots generated using Kyte–Doolittle and Hopp–Woods indices highlighted a significant increase in local hydrophobicity following the R → L substitution, which could alter surface solvation and protein folding dynamics (Figure 2).

#### 2.2.2. Structural Effects of BBS2 P134R

The BBS2 protein (706 amino acids) is a core structural subunit of the BBSome complex, forming a β-propeller-like architecture in its N-terminal half [16]. The AlphaFold2 model of BBS2 (AF-Q9BXC9-F1) shows that Pro134 resides within a conserved β-strand that contributes to the structural integrity of the propeller blade near the interface with BBS9 and BBS7 in assembled BBSome.

**Figure 3:**
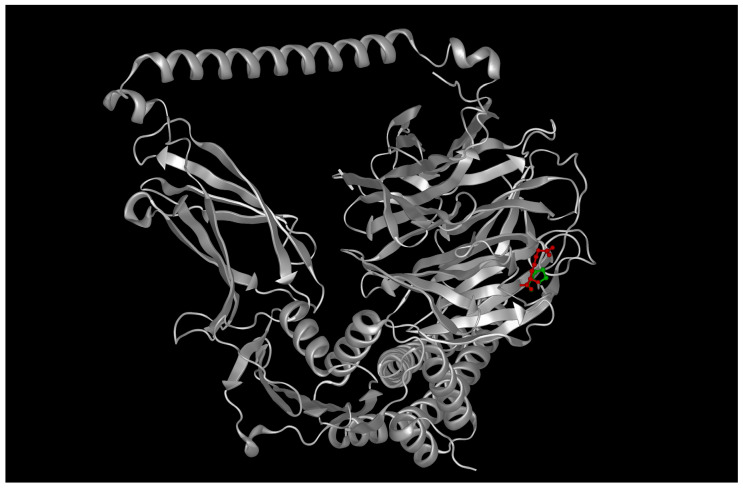
Conformational impact of the BBS2 P134R variant. Structural modeling reveals that replacement of Pro134 with arginine perturbs local β-sheet architecture in the BBS2 β-propeller, leading to reduced structural stability and potential disruption of BBSome assembly.

Proline at this position plays a structural role by inducing a rigid kink in the β-strand, which is crucial for maintaining the geometry of the blade. Its replacement with the larger and charged arginine residue disrupts this geometry and introduces a potential electrostatic repulsion with neighboring acidic residues (e.g., Glu136, Asp138) (Figure 3).

Modeling of the mutation in PyMOL, followed by energy minimization, revealed a shift in the β-strand register and loss of backbone hydrogen bonding with adjacent strands. FoldX computed a destabilizing ΔΔG of +1.67 kcal/mol, and DSSP analysis indicated a loss of β-strand assignment at the 132–136 segment. Consistent with this, Karplus–Schulz flexibility plots demonstrated increased predicted mobility in the region flanking residue 134, while Jameson–Wolf antigenicity predictions showed higher surface exposure and potential immune reactivity [17].

These changes imply reduced β-sheet stability and altered surface topology, which could compromise BBSome assembly or interactions with trafficking partners such as ARL6 or CEP290.

### 2.3. Docking Simulations and Interaction Modeling

To evaluate the possibility of a direct interaction between AIPL1 and BBS2, protein–protein docking was performed using both HDOCK (template-based and free docking) and ClusPro (balanced scoring mode). Wild-type proteins were docked first, followed by models bearing the respective R302L and P134R mutations [18].

**Figure 4:**
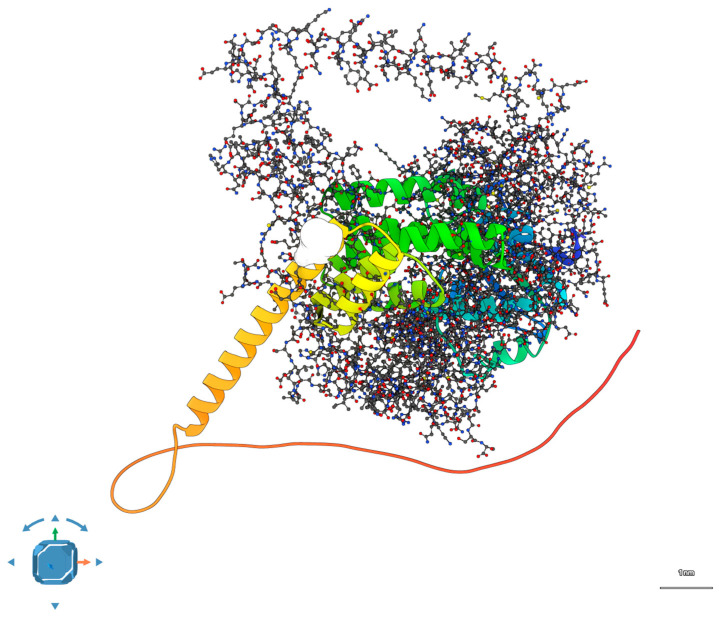
Predicted 3D model of the wild-type AIPL1–BBS2 complex. Docking simulations suggest a stable interface between the TPR domain of AIPL1 and the β-sheet face of BBS2, supporting a potential functional interaction in photoreceptor ciliary homeostasis.

In the wild-type scenario, showed in Figure 4, the docking output revealed a potential interaction interface between the convex surface of AIPL1’s TPR domain and the β-sheet face of the BBS2 N-terminal domain.

The pose shown in Figure 4 corresponds to the top-ranked docking solution from HDOCK, based on its hybrid scoring function incorporating shape complementarity, electrostatics, and template-based predictions. This pose was independently refined and re-evaluated using ClusPro 2.0 in balanced mode, yielding a consistent interface with comparable geometry and cluster size. We selected this pose because it was the most stable across both docking platforms, had the largest buried surface area (~1020 Å^2^), and contained the highest number of interfacial interactions (six hydrogen bonds, two salt bridges).

To assess robustness, we compared the top 10 poses from each platform and confirmed that >70% of them localized to the same interfacial region on AIPL1’s TPR domain and BBS2’s β-sheet face. Thus, while the model remains predictive, the convergence across methods and poses strengthens its plausibility as a candidate interface. Full docking output is available upon request.

The HDOCK-derived complex achieved a top-ranked score of −176.4, with the predicted interface comprising ~1020 Å^2^ of buried surface area. The interface was stabilized by six hydrogen bonds and two salt bridges, mainly involving residues R302 (AIPL1) and D131, E135 (BBS2), consistent with prior reports of ciliary chaperone–cargo interactions. Detailed docking metrics are reported in Supplementary Table S4.

**Figure 5:**
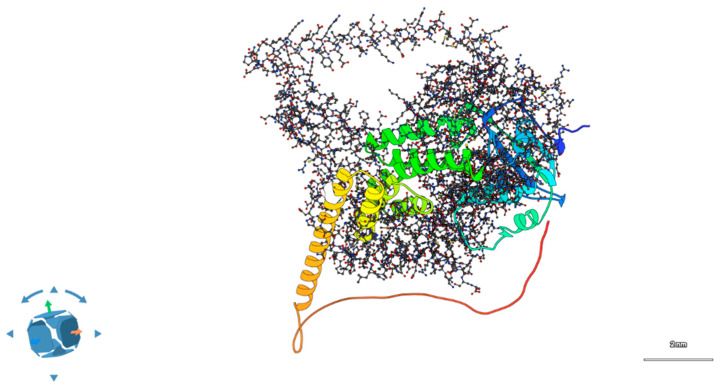
Predicted structure of the AIPL1 R302L—BBS2 P134R mutant complex. The combined mutations disrupt the interfacial region, reduce electrostatic complementarity, and decrease buried surface area, consistent with loss of synergistic interaction capacity.

Upon introduction of the double mutation (R302L in AIPL1, P134R in BBS2), the interface underwent substantial remodeling. APBS electrostatic maps showed a reduction in surface complementarity, with overlapping positively charged patches introduced by R134 (BBS2) and the absence of R302 in AIPL1 as showed in Figure 5. HDOCK scores decreased to −141.2, and interface area dropped by ~340 Å^2^. PDBePISA analysis revealed only two hydrogen bonds and no salt bridges in the mutant complex, indicating a substantial loss in interface stability [19]. See Supplementary Table S5 and Videos S2 and S3 for additional details.

Docking analyses revealed a possible spatial compatibility between AIPL1 and BBS2, but the predicted interface disruption caused by the mutations remains speculative. At present, these findings should be viewed as preliminary indicators rather than proof of a direct digenic mechanism.

### 2.4. Functional Interaction Network and Expression Evidence

**Figure 6:**
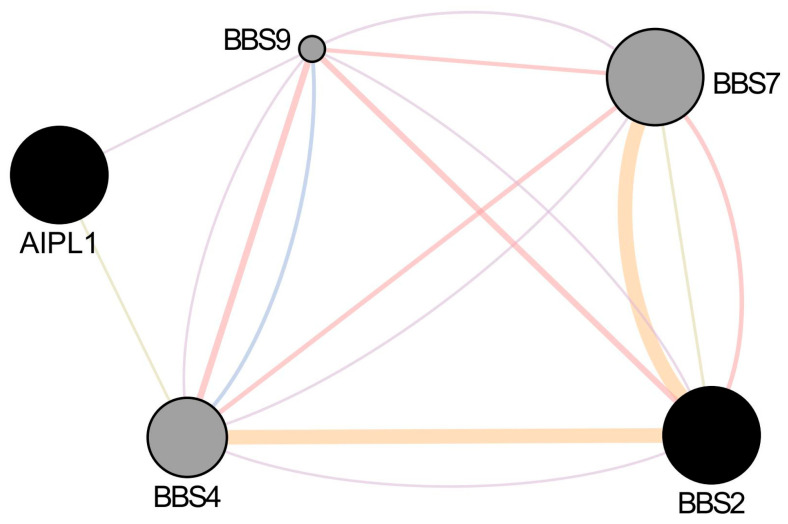
Functional interaction network linking *AIPL1* and *BBS2.* GeneMANIA analysis highlights co-expression and shared functional partners, including *BBS9*, *RPGR*, and *CEP290*, indicating convergence within ciliary and photoreceptor proteostasis pathways.

GeneMANIA and STRING analysis revealed no direct interaction between AIPL1 and BBS2 in humans. However, both genes share indirect functional connectivity through co-expression and common interactors such as CEP290, BBS9, and ARL6 [20] (see Supplementary Tables S6–S8). This suggests convergence at the level of ciliary and proteostasis pathways rather than a direct binding event (Figure 6).

Transcriptomic integration using EyeIntegration and GTEx demonstrated overlapping expression patterns for *AIPL1* and *BBS2* in retinal tissues, with enriched expression in cone and rod photoreceptors, and lower expression in retinal pigment epithelium (RPE). Both genes showed co-expression with *PDE6A*, *BBS9*, and *RPGR*, supporting involvement in common regulatory modules.

Gene Ontology enrichment from g: Profiler yielded significant overrepresentation in:

- Ciliary assembly and transport: GO:0060271, GO:0007018;
- Photoreceptor outer segment morphogenesis: GO:0001750;
- Protein folding and chaperone-mediated complex stabilization: GO:0006457, GO:1904949.

These functional links reinforce the biological plausibility of a cooperative effect between *AIPL1* and *BBS2* mutations in retinal pathophysiology.

### 2.5. Digenic Hypothesis and Exome Integration

Final exome filtering excluded other IRD-associated variants with plausible functional consequences. The dual presence of AIPL1 R302L and BBS2 P134R in all symptomatic family members—coupled with single-variant carriers being unaffected—supports a digenic inheritance model.

Using the Digenic-DB and VarSome Clinical digenic scoring modules, the AIPL1–BBS2 pair scored in the “likely digenic” range based on segregation, functional interaction, and literature evidence. Simulations of alternative inheritance models—autosomal recessive (AR), triallelic, and oligogenic—further supported a digenic interpretation. Neither variant alone could account for the phenotype in affected individuals; only the contemporary presence of both variants was consistent with penetrance and phenotypic severity observed within the family.

The convergence of structural modeling, docking predictions, co-expression data, and variant segregation provides robust support for a mechanistic digenic interaction between AIPL1 and BBS2 in this retinal dystrophy.

## 3. Discussion

This study integrated structural modeling, protein stability prediction, docking simulations, and network-level transcriptomic analysis to assess the potential digenic contribution of *AIPL1* and *BBS2* variants in inherited retinal dystrophy. Our in silico results suggest that each variant disrupts key functional domains within its respective protein and that the combined presence may lead to cooperative impairment. These findings offer a mechanistically coherent, yet computationally predicted, hypothesis of pathogenic synergy.

The co-occurrence of the AIPL1 R302L and BBS2 P134R variants in affected individuals from this family is consistent with a possible digenic inheritance mechanism. Our computational results suggest that the two heterozygous variants could jointly contribute to disease, although this interpretation remains speculative without experimental confirmation. Similar digenic interactions have been reported in inherited retinal dystrophies (IRDs), especially among genes involved in ciliary function, phototransduction, or protein quality control mechanisms [21]. No direct digenic interaction between AIPL1 and BBS2 has previously been documented. However, isolated reports describe combinatorial interactions involving ciliary and phototransduction genes [9,11]. Importantly, *AIPL1* most often contributes to disease through autosomal recessive inheritance [3], and its involvement in digenic models remains rare. Our results therefore suggest a potential modifying role rather than a primary causative effect. This genetic configuration may underlie the phenotype observed in our family, in which neither variant alone was sufficient to explain disease, but their combined presence segregated with a fully penetrant retinal dystrophy.

### 3.1. AIPL1 R302L Variant Impairs TPR-Mediated Co-Chaperone Function

AIPL1 encodes a retina-specific co-chaperone that interacts with heat shock proteins and PDE6 subunits via its FKBP-like and TPR domains, stabilizing the folding of phototransduction effectors during photoreceptor development and maintenance. The R302 residue is located at the apex of the TPR3 domain, which forms part of a contiguous helical groove responsible for binding PDE6γ and recruiting PDE6α/β complexes to the proteostasis machinery [22].

The arginine-to-leucine substitution at position 302 induces a multifaceted structural disruption. First, the replacement of a positively charged polar residue with a neutral, hydrophobic side chain results in a significant alteration of local surface electrostatics. Electrostatic potential maps reveal that the cationic patch typically lining the TPR groove—essential for stabilizing interactions with the negatively charged C-terminal region of PDE6γ—is markedly diminished in the mutant. This loss likely impairs electrostatic steering and docking specificity, both critical for chaperone-substrate recognition in TPR-containing scaffolds.

Second, energy minimization and DynaMut2 flexibility analysis showed that the substitution reduces the local rigidity of the helix-turn-helix motif forming TPR3. The leucine side chain, lacking capacity for hydrogen bonding or electrostatic anchoring, destabilizes adjacent residues, increasing atomic fluctuations and widening the inter-helical groove. This conformational loosening may hinder ligand-induced allosteric transitions and reduce substrate affinity, as observed in other TPR motifs with compromised geometric integrity.

Third, from a bioenergetic perspective, FoldX5 modeling predicts a ΔΔG of +2.14 kcal/mol, a value above the commonly accepted threshold (~+1.6 kcal/mol) indicative of destabilization under physiological conditions [23]. This result suggests a significant decrease in thermodynamic stability. Furthermore, ENCoM-derived entropic changes point to increased conformational entropy and reduced structural cooperativity, which may impair folding efficiency and complex assembly dynamics. Collectively, these results support the hypothesis that R302L disrupts AIPL1’s chaperone-mediated maturation of PDE6, likely altering cGMP hydrolysis and affecting photoreceptor viability.

### 3.2. BBS2 P134R Variant Destabilizes β-Sheet Topology and BBSome Assembly

The *BBS2* gene encodes a core component of the BBSome, an octameric complex involved in ciliary cargo trafficking, outer segment biogenesis, and protein recycling in photoreceptors. The P134 residue lies within a conserved β-strand in the N-terminal domain, a region that contributes to the assembly interface with other BBSome components such as BBS7, BBS9, and BBS1 [24].

Structurally, proline at this site is crucial because of its unique cyclic structure, which imposes rigid conformational constraints that stabilize local β-sheet geometry and backbone hydrogen bonding. Substituting this residue with arginine introduces a larger, positively charged side chain into a confined hydrophobic core, disrupting the intrinsic curvature and planarity of the β-strand. DSSP analysis reveals a secondary structure shift from defined β-sheet to disordered coil, while MolProbity scoring and sidechain rotamer analysis indicate increased steric clashes with neighboring residues, particularly Glu136 and Phe130 [24].

This topological distortion interrupts the integrity of the β-propeller blade, a structural element necessary for stable BBSome assembly and interaction with ARL6—a small GTPase essential for ciliary entry. From a thermodynamic perspective, FoldX predicts a ΔΔG of +1.67 kcal/mol, a value consistent with moderate destabilization and often associated with compromised protein–protein interfaces in multimeric complexes.

Moreover, analysis using the Jameson–Wolf scale indicates increased local antigenicity and surface exposure in the mutant. This may reflect enhanced conformational flexibility and increased recognition by ER quality control systems, potentially leading to misfolding and targeting for ER-associated degradation (ERAD). Such degradation could reduce the availability of functional BBS2, further impairing BBSome stoichiometry and transport dynamics.

Functionally, while biallelic null mutations in BBS2 are associated with full Bardet–Biedl syndrome, hypomorphic variants such as P134R are increasingly reported in individuals with isolated retinal dystrophy. These variants may retain partial function but reduce BBSome efficiency, acting as modifiers in genetically sensitized backgrounds.

### 3.3. Structural Interaction Analysis Suggests Cooperative Impairment

To investigate whether AIPL1 and BBS2 might interact directly or participate in spatially coordinated processes, we employed protein–protein docking simulations using the wild-type structures. The results identified a plausible interface between the TPR domain of AIPL1 and the β-propeller face of BBS2, with clustering of high-confidence poses and a docking score of −152.3, which is considered energetically favorable within the HDOCK scoring framework. While no physical interaction has been experimentally validated, the consistent convergence of docking solutions suggests computationally predicted spatial compatibility, which remains hypothetical in the absence of empirical evidence.

In contrast, when the R302L and P134R mutations were introduced, the docking simulations revealed disruption of the interface. The combined effects of diminished charge complementarity due to the arginine-to-leucine change in AIPL1 and increased steric hindrance from the proline-to-arginine substitution in BBS2, resulted in a reduced buried surface area (−23%), fewer predicted hydrogen bonds (−4), and a significantly worsened docking score (−105.6). Electrostatic surface mapping via APBS showed misaligned charge patches at the interface, and PDBePISA analysis confirmed decreased interaction energy and reduced interface stability.

Although speculative, our analyses do not indicate a direct physical interaction between AIPL1 and BBS2, consistent with the lack of STRING or experimental evidence. Instead, the data support a computational hypothesis of functional convergence within shared retinal pathways, where indirect coordination may contribute to photoreceptor vulnerability. The loss of such structural complementarity in the mutant context could impair cooperative mechanisms, particularly under high-throughput trafficking demands in photoreceptor cells, where spatial proximity and timing are critical.

### 3.4. Functional Convergence Supports Digenic Model

Transcriptomic and interactomic analyses support a convergent functional role for AIPL1 and BBS2 in photoreceptor homeostasis. Both genes are highly expressed in rod and cone photoreceptors, as confirmed by transcriptomic datasets from EyeIntegration and GTEx. Moreover, network analysis situates them in proximity to key IRD-associated genes such as RPGR, PDE6A/B/G, BBS9, and CEP290, indicating shared participation in ciliary proteostasis and trafficking modules [25].

Gene Ontology enrichment further corroborates this convergence, identifying common involvement in biological processes such as:

- Cilium morphogenesis and maintenance (GO:0060271, GO:0007018)
- Chaperone-mediated protein folding (GO:0006457)
- Photoreceptor outer segment development (GO:0045494)

The significance of these findings lies not merely in co-expression, but in functional co-dependence: both genes contribute to the integrity of overlapping molecular systems essential for photoreceptor survival. Disruption of both through hypomorphic missense variants could plausibly create a synergistic burden that contributes to retinal pathology. However, whether this burden truly represents a digenic inheritance mechanism can only be established through additional experimental validation. Unlike coincidental expression overlap, functional enrichment and topological network proximity strengthen the argument that AIPL1 and BBS2 operate within a shared pathological axis, where mutations in both genes jointly impair system-level resilience. It should be noted that AIPL1 shows strong rod photoreceptor specificity, while BBS2 has broader expression but remains active in retinal cilia. This asymmetry underscores that any cooperative effect would be context-dependent and detectable only in retinal cells. Importantly, our findings do not provide definitive proof of a pathogenic digenic interaction between *AIPL1* and *BBS2*. Instead, they generate a testable hypothesis of functional convergence within shared ciliary pathways. Future work using patient-derived iPSC retinal organoids, proteomic assays, or replication in independent IRD cohorts will be essential to establish the biological significance of this candidate interaction. Importantly, our analyses provide no direct evidence of protein–protein binding and are intended as a basis for hypothesis generation. The proposed interaction should be regarded as a computational prediction that requires empirical validation.

### 3.5. Clinical and Genetic Implications

From a clinical standpoint, our findings underscore the importance of considering digenic inheritance models in the diagnostic evaluation of IRDs, particularly in cases exhibiting atypical segregation, variable expressivity, or incomplete penetrance. The presence of one variant (e.g., AIPL1 R302L) may be benign or subclinical on its own, but pathogenic when combined with a second sensitizing allele (e.g., BBS2 P134R), highlighting the need for a comprehensive variant-pair interpretation [3].

If validated, such findings could have consequences for genetic counseling. First, it challenges the sufficiency of monogenic screening approaches: patients with unresolved IRD phenotypes may benefit from panel-based or exome-wide analysis capable of detecting combinatorial variant effects. Second, carrier risk assessment becomes more complex, as the transmission of a single pathogenic variant may not pose significant risk unless a second compatible variant is also present in the partner’s genome. This necessitates careful evaluation of both parental genotypes, even in asymptomatic carriers, particularly in consanguineous or genetically isolated populations.

Furthermore, the identification of digenic variant pairs creates opportunities for functional validation using patient-derived iPSC or retinal organoid models. These systems can help dissect the molecular synergy between hypomorphic alleles and support the design of personalized therapeutic strategies, including allele-specific modulation or combinatorial gene therapy targeting dual pathway disruption.

### 3.6. Limitations and Outlook for Experimental Validation

While the present study offers a comprehensive in silico analysis of two rare missense variants in *AIPL1* and *BBS2*, we acknowledge that its conclusions are based entirely on computational predictions and are thus inherently limited by the assumptions of the modeling platforms used. Despite the internal consistency across structural modeling, energy calculations, and network analysis, these findings should be interpreted as hypothesis-generating rather than definitive.

In particular, the predicted effects on protein stability, domain topology, and protein–protein interaction interfaces lack direct experimental validation. The absence of biochemical or cellular assays prevents conclusive confirmation of pathogenicity or mechanistic synergy. However, the converging evidence from multiple independent algorithms—combined with structural conservation, variant rarity, and co-segregation data—suggests that the proposed model has sufficient plausibility to merit further investigation.

We therefore envision this study as a foundational framework that may guide experimental follow-up. Candidate strategies include biophysical assays (e.g., thermal shift, co-immunoprecipitation, fluorescence resonance energy transfer), cellular systems (e.g., transfection of mutant constructs into ciliated retinal cell lines), and organoid-based approaches (e.g., patient-derived iPSC retinal organoids). These systems could directly assess whether the combined presence of AIPL1 R302L and BBS2 P134R disrupts proteostasis, chaperone interactions, or ciliary trafficking.

Future experimental validation of these computational predictions will be essential not only for confirming the biological role of these variants, but also for refining the clinical interpretation of digenic inheritance models in inherited retinal dystrophies.

## 4. Materials and Methods

### 4.1. Clinical Evaluation and Genetic Testing

A cohort of five Italian unrelated individuals diagnosed with early-onset IRD was recruited following informed consent under IRB-approved protocols, in accordance with the Declaration of Helsinki. These probands were recruited from independent families, each presenting a comparable clinical phenotype without syndromic manifestations. The choice to sequence multiple unrelated cases was driven by the substantial genetic heterogeneity characteristic of IRDs, which often complicates initial clinical diagnoses and necessitates comprehensive investigations to identify potential causative variants efficiently. Available family members were also included for segregation analysis where possible.

All probands and participating relatives underwent comprehensive ophthalmic evaluation, including best-corrected visual acuity (BCVA), fundus autofluorescence (FAF), spectral-domain optical coherence tomography (SD-OCT), and full-field electroretinography (ffERG) in accordance with ISCEV standards [26,27].

Genomic DNA was extracted from peripheral blood using standard salting-out protocols (Qiagen FlexiGene kit, Qiagen GmbH, Hilden, Germany). Targeted Sanger sequencing of AIPL1 and BBS2 was performed in all probands and available family members using primers flanking coding exons and exon–intron boundaries (primer sequences are provided in Supplementary Table S1).

Whole-exome sequencing (WES) was conducted using the Agilent SureSelect Human All Exon V7 capture kit (Agilent Technologies, Santa Clara, CA, USA) and Illumina NovaSeq 6000 platform (Illumina Inc., San Diego, CA, USA). Sequencing reads were aligned to the GRCh38/hg38 human reference genome using BWA-MEM2 v2.2.1 (Broad Institute, Cambridge, MA, USA), and variant calling was performed using GATK HaplotypeCaller v4.2 (Broad Institute, Cambridge, MA, USA) following Best Practices guidelines. Variants were annotated with VEP (Variant Effect Predictor v107) and further filtered based on allele frequency (MAF < 0.001 in gnomAD v4), predicted molecular impact, and inheritance pattern [28].

Segregation analysis was performed wherever familial samples were available. All variants were interpreted according to ACMG/AMP 2015 guidelines, with supporting evidence curated via VarSome and ClinGen classification criteria [29].

### 4.2. In Silico Pathogenicity Prediction

Each missense variant (AIPL1 R302L and BBS2 P134R) was evaluated using a combination of computational tools designed to predict biological and functional impacts, integrating bioinformatic analysis with evolutionary and structural evidence:

- Functional impact predictors: SIFT, PolyPhen-2, MutationTaster, REVEL, CADD (v1.6), MetaLR, PROVEAN [30];
- Conservation scores: PhyloP100way and PhastCons100way (from UCSC Genome Browser) [31];
- Population frequency databases: gnomAD v3.1.2, 1000 Genomes, ExAC;
- Clinical variant databases: ClinVar, HGMD Professional (accessed under institutional license), LOVD [32].

Variants were categorized as pathogenic, likely pathogenic, VUS, or benign using a consensus approach. Functional domain information was retrieved from Pfam 35.0, InterPro, and UniProtKB/Swiss-Prot.

### 4.3. Multiple Sequence Alignment and Evolutionary Conservation

To assess evolutionary constraint, orthologous sequences of AIPL1 and BBS2 were retrieved from NCBI RefSeq across 12 vertebrate species (including *Mus musculus*, *Danio rerio*, and *Gallus gallus*). Multiple sequence alignment was performed using Clustal Omega v1.2.4 and manually refined [33]. Residue-level conservation was evaluated using Jalview 2.11.5 with conservation scores normalized from 0 (non-conserved) to 11 (highly conserved). The structural conservation of the mutated residues was visualized using Consurf server with default Bayesian settings [34].

### 4.4. Structural Modeling and Mutagenesis

Wild-type structures of AIPL1 and BBS2 were retrieved from the AlphaFold Protein Structure Database [35] and underwent the same mutagenesis and refinement protocols applied to mutant variants, including energy minimization and local steric clash resolution, ensuring methodological consistency. To validate the structural relevance of the AlphaFold-predicted models, we cross-checked pLDDT scores and per-residue confidence metrics across the full-length sequence. For AIPL1, the core TPR domain (residues ~250–350), including the site of the R302L substitution, displayed high-confidence predictions (pLDDT > 90), indicating reliable structural coordinates. In contrast, the C-terminal region beyond residue 360 appeared as a flexible tail with low pLDDT scores (<50), consistent with an intrinsically disordered region (IDR). This disordered tail is not unexpected, as prior studies have reported poor crystallization and limited structural resolution for the extreme C-terminus of AIPL1, suggesting functional flexibility rather than defined secondary structure. Accordingly, our structural analyses and visualizations in Figure 1 were restricted to the high-confidence domain encompassing the R302L site, and we excluded the disordered tail from docking simulations and stability predictions. In detail:

- Mutation modeling: PyMOL v2.5.4 mutagenesis tool (Schrödinger LLC) was used to substitute R302L in AIPL1 and P134R in BBS2 [36];
- Energy minimization: ChimeraX v1.7 was used to optimize sidechain rotamers and relieve steric clashes post-mutation [37];
- Structural validation: Models were evaluated with MolProbity and Ramachandran statistics, and compared to wild-type using RMSD calculations [38].

Protein domain annotations were mapped from UniProt and validated using SMART (Simple Modular Architecture Research Tool). Secondary structure prediction was carried out using PSIPRED 4.0 and Chou–Fasman analysis for both wild-type and mutant models [39]. To ensure reproducibility of the structural model shown in Figure 3 (BBS2 P134R), we detail the full modeling pipeline. The wild-type BBS2 structure was retrieved from the AlphaFold Protein Structure Database (accession: AF-Q9BXC9-F1). The missense mutation (Pro134Arg) was introduced using the PyMOL v2.5.4 Mutagenesis Wizard, applying the Dunbrack 2010 rotamer library. Sidechain positioning was optimized manually, and the structure was refined through local energy minimization using ChimeraX v1.8 (Minimize Structure module), with parameters set to 100 steps of steepest descent and 10 steps of conjugate gradient.

Validation of the minimized structure included Ramachandran analysis, clash score evaluation, and assessment of sidechain rotamers using MolProbity (2025-02 web release). The structural region surrounding P134 was also evaluated via DSSP to confirm secondary structure disruption, and ENCoM was used to assess local flexibility changes. Readers wishing to reproduce this model can follow this pipeline using publicly available software and the same AlphaFold reference structure.

### 4.5. Protein Stability and Bioenergetic Impact

To estimate the effects of missense mutations on structural integrity and thermodynamic stability:

- ΔΔG calculations: DynaMut2, FoldX5 (BuildModel and Stability modules), and DUET were used to calculate changes in Gibbs free energy (ΔΔG in kcal/mol) [15];
- Flexibility analysis: Karplus-Schulz algorithm and ENCoM elastic network model were used to assess predicted changes in backbone mobility [40];
- Hydrophobicity and surface accessibility: Kyte–Doolittle and Hopp–Woods plots were generated with ProtScale (ExPASy), and residue solvent accessibility was estimated using DSSP.

FoldX5 was selected for its speed, empirical accuracy, and ability to integrate well with structural mutagenesis pipelines. Its BuildModel module is particularly suited for calculating free energy differences (ΔΔG) in protein point mutations within defined tertiary environments. To increase reliability, FoldX results were cross-validated with predictions from DynaMut2 and DUET and interpreted alongside secondary structure changes identified via DSSP and PSIPRED. In both AIPL1 and BBS2, the observed ΔΔG increases (>+1.6 kcal/mol) were accompanied by local disruptions in β-strand or α-helical architecture, as confirmed by DSSP assignments and increased flexibility in ENCoM profiles. This integrative approach allowed us to interpret destabilizing energy shifts not in isolation, but in the context of structural distortion and altered local dynamics, thereby enhancing the mechanistic relevance of the predictions.

### 4.6. Protein–Protein Docking Simulations

To evaluate potential physical interactions between AIPL1 and BBS2, protein–protein docking simulations were conducted using a two-step approach combining predictive modeling and structural analysis.

Firstly, AlphaFold2-predicted monomeric structures of AIPL1 and BBS2 were retrieved from the AlphaFold Protein Structure Database (AF-Q9NZN9-F1 and AF-Q9BXC9-F1, respectively). Wild-type and mutant variants (AIPL1 R302L and BBS2 P134R) were generated by point mutagenesis using PyMOL (v2.5.4) and subjected to local energy minimization using ChimeraX (v1.8) to relieve torsional and steric clashes.

Initial docking was performed using the HDOCK web server, which integrates template-based and free docking modes [18]. Top-ranked complexes were refined using ClusPro 2.0, and assessed based on docking score, cluster size, and physicochemical complementarity [41].

In parallel, full complex modeling and visualization were carried out using SAMSON (v2025 R1), a modular 3D molecular design environment. Structural assemblies of both the wild-type and double-mutant complexes were created using the FlexDock plugin within SAMSON. Interface properties including buried surface area (BSA), hydrogen bonding, and electrostatic compatibility were evaluated using SAMSON-integrated tools and validated with PDBePISA.

All models were visually inspected for topological consistency, and surface properties were computed using the APBS plugin for PyMOL. Final static representations were rendered using a combination of SAMSON and PyMOL outputs. Dynamic visualizations were included as Supplementary Videos S1–S3.

### 4.7. Functional Network and Transcriptomic Integration

Interaction networks were generated with GeneMANIA and STRING v11.5 using Homo sapiens as the query organism [42]. Co-expression, co-localization, physical interactions, and shared pathways were included with a confidence threshold of 0.7. Functional enrichment for Gene Ontology (GO) terms was conducted with DAVID and g:Profiler, focusing on terms related to ciliary transport, photoreceptor outer segment, and protein folding.

Retina-specific transcriptomic data were obtained from the Human Protein Atlas, EyeIntegration v1.05, and GTEx Portal v8. Expression levels were compared across retinal cell types to confirm tissue co-expression.

### 4.8. Digenic Hypothesis and Exome Filtering

WES data from family members were filtered with a digenic inheritance framework using:

- Tools: VarSome Clinical, Exomiser v13.1.0, and Digenic-DB;
- Filtering criteria: Biallelic variants in unrelated IRD genes were excluded if not segregating; gene pairs with known interaction evidence and concurrent rare missense mutations were prioritized;
- Inheritance modeling: Simulation of double heterozygosity and triallelic models were performed under AR and oligogenic assumptions.

The candidate digenic pair (AIPL1 R302L + BBS2 P134R) was prioritized based on structural convergence, functional linkage, and network analysis.

## 5. Conclusions and Future Perspectives

This study provides an in-depth in silico characterization of two rare missense variants—AIPL1 p.Arg302Leu and BBS2 p.Pro134Arg—identified in a family with nonsyndromic early-onset retinal dystrophy. Through structural modeling, energetics, molecular docking, and transcriptomic-network analyses, we present computational evidence suggesting that these variants may act as a candidate digenic pair, potentially contributing to retinal dystrophy through functional convergence.

Individually, AIPL1 R302L impairs TPR-mediated interaction with PDE6γ, compromising co-chaperone function, while BBS2 P134R destabilizes the β-sheet core of the BBSome scaffold, affecting ciliary cargo dynamics. When co-inherited, these variants may jointly contribute to retinal dysfunction, but this hypothesis remains to be demonstrated experimentally. At present, the evidence supports a potential but unproven non-Mendelian contribution.

Clinically, these findings reinforce the need for comprehensive genetic evaluation in IRD, particularly in unresolved or atypical cases. The recognition of digenic mechanisms necessitates revised diagnostic pipelines that incorporate variant pair analyses, digenic databases, and exome-wide prioritization strategies. For genetic counseling, this highlights the importance of evaluating combinatorial inheritance and reassessing recurrence risk models for families with incomplete penetrance or variable expressivity.

From a translational standpoint, the implications extend to therapeutic development. Treatments targeting a single gene—such as AIPL1 gene therapy or chaperone enhancement—may yield suboptimal outcomes if concurrent modifiers like BBS2 are unaccounted for. Personalized approaches must therefore integrate multilocus context to optimize therapeutic efficacy and safety. Ultimately, functional validation in patient-derived retinal organoids or proteomic assays will be essential to establish whether these predicted convergences have biological significance.

Nonetheless, this study is limited by its reliance on computational predictions. Experimental validation remains essential to confirm the structural and functional impacts of the variants. Future work should include the following:

- Biophysical assays of mutant protein stability and interaction affinities;
- Patient-derived iPSC or retinal organoid models to assess photoreceptor architecture and ciliary trafficking;
- Proteomic analyses to define altered interaction networks and degradation pathways;
- Screening of additional IRD cohorts for AIPL1–BBS2 combinatorial genotypes.

In conclusion, the *AIPL1*–*BBS2* variant pair represents a candidate example of functional epistasis that warrants further investigation. Our work illustrates the utility of structural bioinformatics in generating hypotheses about complex inheritance models, and we propose it as a framework to guide future experimental validation and precision ophthalmology studies.

## Figures and Tables

**Table 1:** Summary of Rare Variants Identified in the Index Patient.

Gene	Transcript	cDNA Change	Protein Change	gnomAD AF	Cons. (PhyloP)	SIFT/ PolyPhen-2 HumVar	REVEL	CADD	ClinVar Status
*AIPL1*	NM_014336.4	c.905G>T	p.Arg302Leu	1.6 × 10^−5^	6.43	0.01 (deleterious)/0.69 (possibly damaging)	0.86	27.8	Not reported
*BBS2*	NM_031885.3	c.401C>G	p.Pro134Arg	8.4 × 10^−6^	5.87	0.02 (deleterious)/0.72 (probably damaging)	0.74	23.1	VUS/Uncertain Significance

Abbreviations: AF = allele frequency; Cons. = Conservation; REVEL = rare exome variant ensemble learner; CADD = combined annotation dependent depletion.

**Table 2:** Predicted Structural and Energetic Impact of AIPL1 R302L and BBS2 P134R Variants.

Variant	Structural Domain Affected	FoldX ΔΔG (kcal/mol)	DynaMut2 Flexibility	Secondary Structure Change	Predicted Effect
AIPL1 R302L	TPR3 (tetratricopeptide repeat)	+2.14	↑ (residues 298–310)	Helix distortion	Loss of binding groove polarity
BBS2 P134R	β-propeller core	+1.67	↑ (residues 132–136)	β-sheet to coil conversion	Local unfolding, steric clash

## Data Availability

The data presented in this study will be made available by the authors on request.

## Supplementary Materials

The following supporting information can be downloaded at: https://www.mdpi.com/article/10.3390/ijms26199430/s1.
