# Supplementary material for: Computational Evidence for Digenic Contribution of AIPL1 and BBS2 Rare Variants in Inherited Retinal Dystrophy
Source: Int J Mol Sci. 2025 Sep 26;26(19):9430. doi: 10.3390/ijms26199430 (PMC12524372; doi:10.3390/ijms26199430)
Supplement: Supplementary file 1 [file ijms-26-09430-s001.zip › Supplementary Tables and Videos.pdf]

Supplementary Tables

Supplementary Table S1. Primer sequences used for Sanger sequencing of *AIPL1* and *BBS2* coding exons.

Gene	Variant (amplicon, GRCh38)	Amplicon size (bp)	Primer	Sequence (5′→3′)
AIPL1	p.R302L (chr17:6425523–6425919)	397	AIPL1_F	TGCGGACAGCTCTGCAGATG
			AIPL1_R	GTCTTGGAGGCTGGTGAGTC
BBS2	p.P134R (chr16:56510995–56511424)	430	BBS2_F	GAGAGGATTACACAAAACACG
			BBS2_R	AATGTGTGAGGCCCACCCAC

Supplementary Table S2. Electrostatic Surface Potential and Charge Shift at Mutation Sites

Protein	Residue Change	APBS ΔΦ (kT/e)	Surface Charge Shift	Functional Implication
**AIPL1**	R302 → L	−12.3	Positive → neutral	Loss of PDE6γ interaction groove polarity
**BBS2**	P134 → R	+8.7	Neutral → positive	Charge repulsion near acidic residues

Supplementary Table S3. Electrostatic Potential Shift at Mutation Sites (APBS)

Protein	Residue Change	ΔΦ (kT/e) @ Site	Surface Charge Shift	Functional Interpretation
**AIPL1**	R302 → L	−12.3	Positive → Neutral	Loss of positive groove for PDE6γ
**BBS2**	P134 → R	+8.7	Neutral → Positive	Electrostatic clash at β-interface

Supplementary Table S4. Docking Score and Interface Metrics (HDOCK + SAMSON)

Complex Configuration	Docking Tool	Docking Score	Cluster Size	Interface Area (Å²)	H-Bonds	Salt Bridges	ΔBSA (vs WT)
**WT AIPL1 + WT BBS2**	HDOCK	−176.4	42	1020	6	2	—
**AIPL1 R302L + BBS2 P134R**	HDOCK	−141.2	18	680	2	0	−33%
**WT AIPL1 + BBS2 P134R**	HDOCK	−163.1	29	860	4	1	−16%
**AIPL1 R302L + WT BBS2**	HDOCK	−150.2	21	740	3	1	−27%

Supplementary Table S5. AIPL1–BBS2 Complex Properties (SAMSON Analysis)

Complex Configuration	BSA (Å²)	Interface RMSD (Å)	Interaction Hotspots	Visual Stability (Video)	Structural Outcome
**WT**	1020	—	Arg302 (AIPL1), Glu196 (BBS2)	Supplementary Video S2	Stable interface
**Double mutant (R302L+P134R)**	680	2.6	Leu302, Arg134 → clash	Supplementary Video S3	Interface deformation

Supplementary Table S6. Expression and Co-expression Profile of AIPL1 and BBS2 (EyeIntegration & GTEx)

Gene	Retina (TPM)	RPE (TPM)	Rods (log2FC)	Cones (log2FC)	Co-expression Partners (r > 0.8)
**AIPL1**	87.3	15.6	+3.8	+3.1	*PDE6A, RPGR, BBS9*
**BBS2**	42.1	10.3	+2.6	+2.0	*CEP290, BBS9, ARL6, IFT172*

Supplementary Table S7. Summary of Pathway Enrichment for AIPL1 and BBS2 Shared Interactors

Enriched GO Term	GO ID	Description	Genes Involved	FDR
**Cilium morphogenesis**	GO:0060271	Biogenesis and structure of primary cilium	*AIPL1, BBS2, CEP290, RPGR*	1.3×10^{-4}
**Protein folding**	GO:0006457	Assisted polypeptide folding	*AIPL1, HSP90AA1, DNAJB6*	4.6×10^{-3}
**Photoreceptor outer segment organization**	GO:0001750	Morphology of outer segment of rods/cones	*BBS2, BBS9, PDE6A, CNGB1*	6.1×10^{-4}

Supplementary Table S8. Co-Expression in Retinal Tissues (EyeIntegration v1.1)

Gene	Retina TPM	Rod-specific log2FC	Top Co-Expressed Genes	Shared Pathways
**AIPL1**	87.3	+3.8	*PDE6A, RPGR, BBS9*	Protein folding, phototransduction
**BBS2**	42.1	+2.6	*CEP290, IFT172, ARL6*	Ciliary trafficking

Supplementary Videos

Supplementary Video S1. Rotational animation of the wild-type AIPL1 protein structure highlighting the TPR domain surface topology.

Supplementary Video S2. Comparison of the wild-type and R302L mutant AIPL1, showing changes in electrostatic surface potential.

Supplementary Video S3. Dynamic visualization of BBS2 P134R variant highlighting distortion in the β-sheet core.
